# An adult case with absence of left pulmonary artery and partial anomalous pulmonary venous connection: Case report and literature review

**DOI:** 10.1002/ccr3.3858

**Published:** 2021-01-25

**Authors:** Sanaz Asadian, Sedigheh Saedi, Bahareh Jahanshahi, Leila Hosseini, Nahid Rezaeian

**Affiliations:** ^1^ Rajaie Cardiovascular Medical and Research Center Iran University of Medical Sciences Tehran Iran; ^2^ North Khorasan University of Medical sciences Bojnurd Iran

**Keywords:** left pulmonary artery, partial anomalous pulmonary venous connection (PAPVC), unilateral absence of pulmonary artery (UAPA)

## Abstract

Unilateral absence of pulmonary artery is a rare congenital disorder that can remain asymptomatic until adulthood. Absence of left pulmonary artery (ALPA) has been reported in one‐third of these patients. We are the first to introduce an adult case of ALPA associated with partial anomalous pulmonary venous connection.

## Graphical abstract

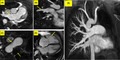

## 1 INTRODUCTION

Unilateral absence of pulmonary artery (UAPA) is a rare congenital disorder that can occur either in isolation or in association with other congenital cardiac disorders such as tetralogy of Fallot, coarctation of the aorta, and atrial septal defect.^1^

Isolated UAPA affects the right lung in two‐thirds of this patient population, with the majority either remaining asymptomatic or having mild clinical symptoms until adulthood. Some patients may develop symptoms such as dyspnea, chest pain, recurrent respiratory infection, and hemoptysis.^2^, ^3^ Diagnosis may be difficult in these patients; the anomaly is often incidentally found on chest radiographs. Chest X‐ray typically shows asymmetric lung fields with ipsilateral hemithorax lucency and mediastinal shift toward the affected side with the ipsilateral absence of hilar vessels.^4^, ^5^ Computed tomography (CT) confers a more desirable depiction of the condition by demonstrating the collateral blood flow, parenchymal mosaic attenuation, and secondary bronchiectasis due to recurrent pulmonary infections. Cardiac magnetic resonance (CMR) imaging is capable of defining anatomic structures and the related functional characteristics, including right ventricular function and strain, concurrently.^4^, ^5^, ^6^

This report presents a rare case of UAPA associated with partial anomalous pulmonary venous connection (PAPVC) and underscores the role of noninvasive imaging modalities through a literature review.

### 1.1 Case history

**FIGURE 1:**
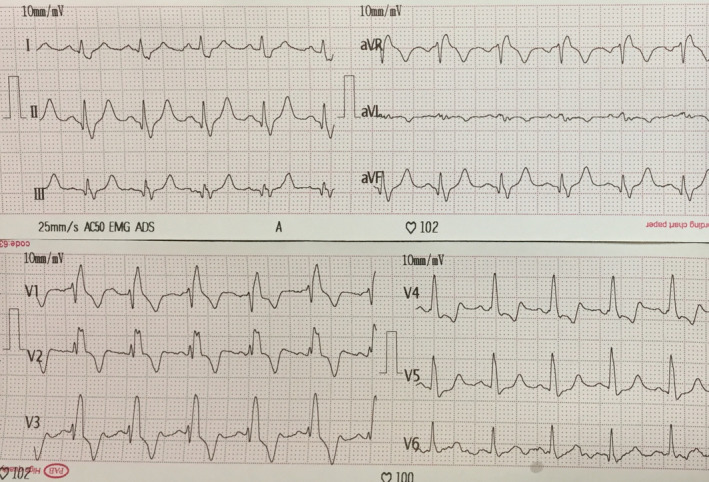
Electrocardiography shows right bundle branch block

**FIGURE 2:**
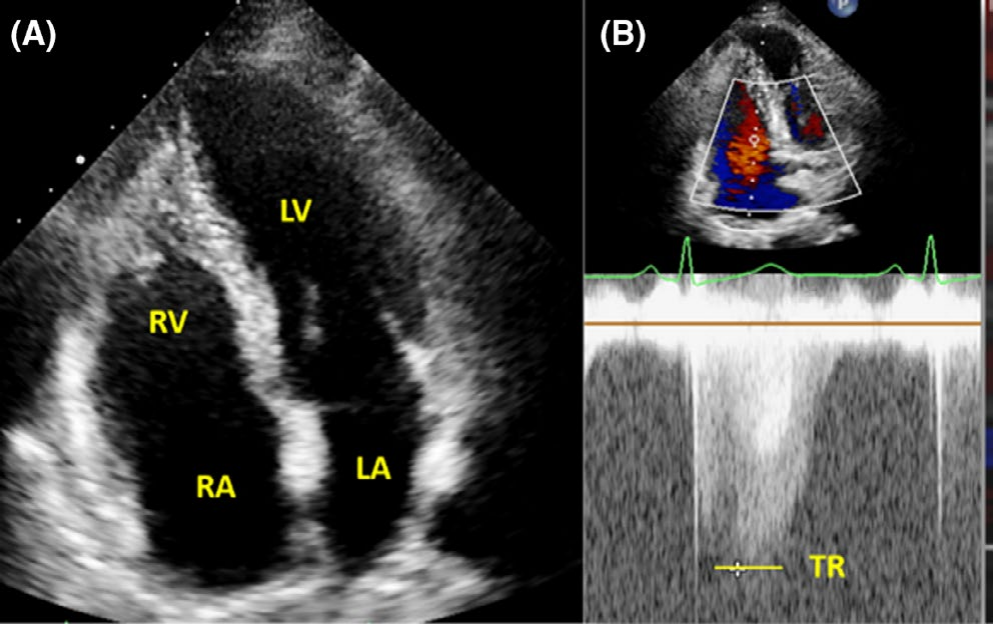
In this echocardiographic image, (A) right ventricular 4‐chamber view shows a mildly enlarged right ventricle and (B) the continuous wave Doppler of the tricuspid jet indicates a mildly increased tricuspid regurgitation velocity

The patient was a 37‐year‐old woman with the chief complaint of occasional palpitations in the preceding few months. She had no history of shortness of breath or fatigue. Her past medical, familial, and drug history was unremarkable. The only notable finding on the initial physical examination was decreased respiratory sounds in the left hemithorax. Electrocardiography illustrated normal sinus rhythm with right bundle branch block (Figure 1). Chest X‐ray demonstrated a hypoplastic left lung with decreased vascular markings, along with leftward mediastinal shift. Transthoracic echocardiography showed mild‐to‐moderate ventricular dysfunction, mild right ventricular enlargement, mild pulmonary hypertension, and possible absence of left pulmonary artery (ALPA) (Figure 2).

### 1.2 Differential diagnosis, investigations, and treatment

**FIGURE 3:**
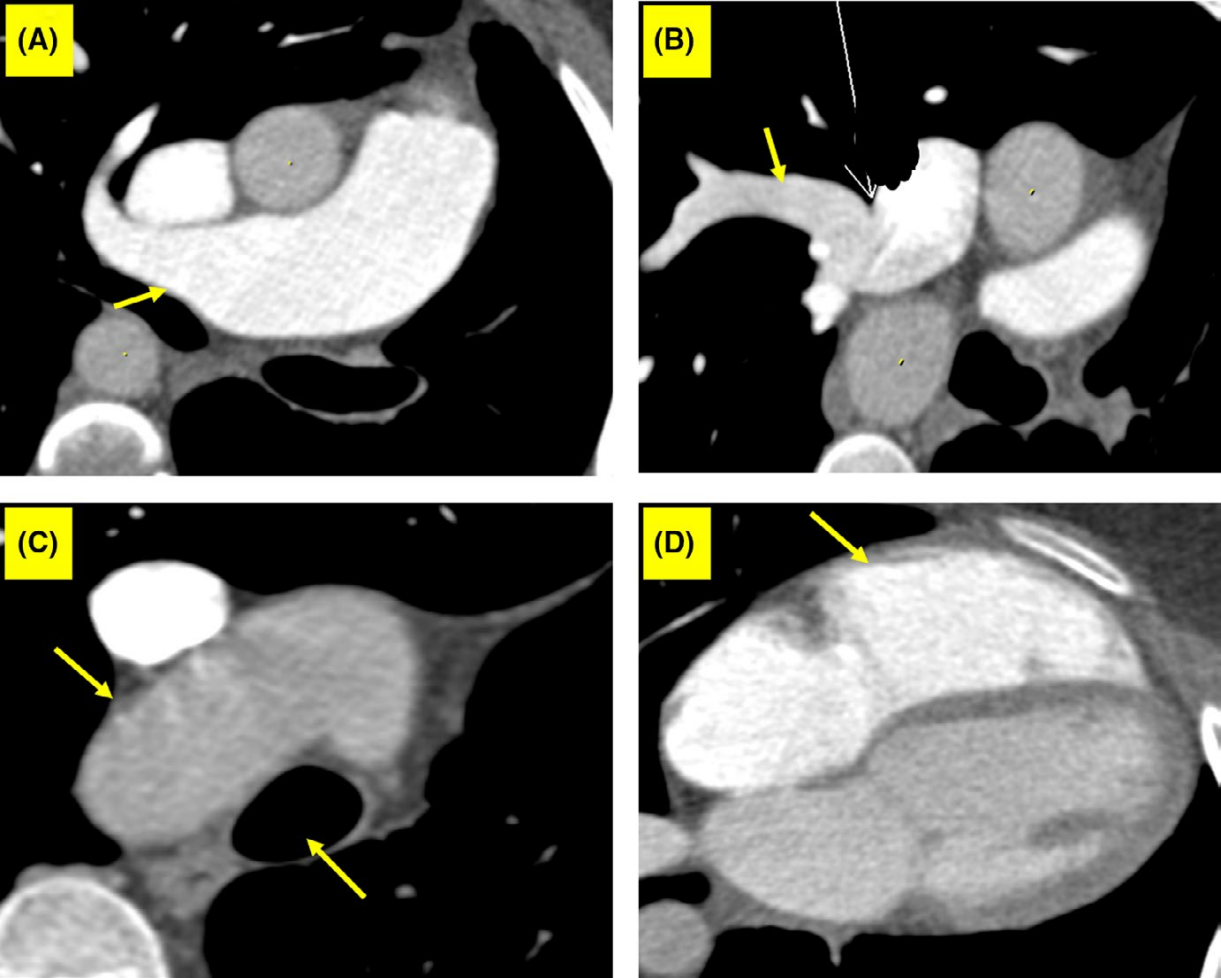
Computed tomography angiography views of the patient with UAPA show (A) absence of LPA with RPA dilation (arrow), (B) PAPVC between RUPV and SVC (arrow), (C) right‐sided aortic arch (upper arrow, with the lower arrow pointing to the trachea), and (D) mild RV enlargement (arrow) in the 4‐chamber view. Abbreviations: UAPA, unilateral absence of pulmonary artery; LPA, left pulmonary artery; RPA, right pulmonary artery; PAPVC, partial anomalous pulmonary venous return; SVC, superior vena cava; RV, right ventricle

Based on the patient's physical examination, chest X‐ray, and transthoracic echocardiography findings, we planned CT angiography to better visualize her anatomic structures. The imaging procedure was performed in 2 consecutive phases: pulmonary arterial enhancement, followed by peak aortic enhancement. The main findings were ALPA, PAPVC between the right upper pulmonary vein (RUPV) and the superior vena cava (SVC), and right‐sided aortic arch (Figure 3).

**FIGURE 4:**
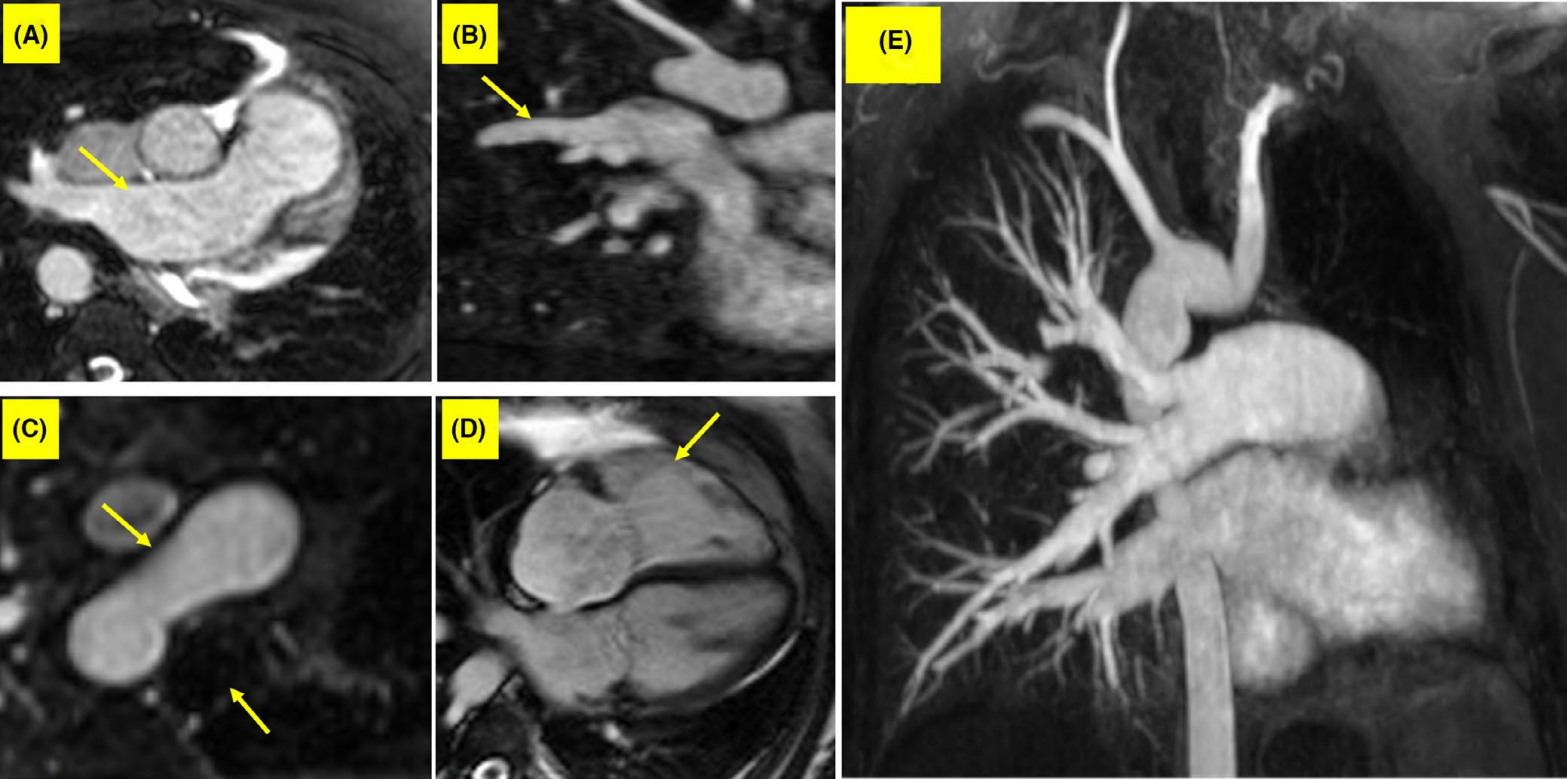
Cardiac magnetic resonance images of the patient with UAPA show (A) absence of LPA with RPA dilation (arrow), (B) PAPVC between RUPV and SVC (arrow), (C) right‐sided aortic arch (upper arrow, with the lower arrow pointing to the trachea), (D) mild RV enlargement (arrow) in the 4‐chamber view, and (E) diminished left lung vascular markings. Abbreviations: CMR, cardiac magnetic resonance; UAPA, unilateral absence of pulmonary artery; LPA, left pulmonary artery; RPA, right pulmonary artery; PAPVC, partial anomalous pulmonary venous return; SVC, superior vena cava; RV, right ventricle

Given the superiority of CMR in the evaluation of right ventricular anatomy and function, we considered it in the next step and found normal left ventricular size with mildly reduced systolic function (ejection fraction = 46%), mild right ventricular enlargement with moderately reduced systolic function (ejection fraction = 37.5%), right‐sided aortic arch, ALPA, dilated main pulmonary artery, dilated right pulmonary artery, PAPVC between RUPV and SVC, diminished left lung vascular markings, and leftward mediastinal shift. Additionally, the phase‐contrast velocity mapping CMR sequence for flow measurement calculated a pulmonary‐to‐systemic flow ratio of 1.6, which represented a significant left‐to‐right shunt (Figure 4).

Because of right ventricular enlargement and significant shunting, the patient underwent a corrective surgical operation for PAPVC.

### 1.3 Outcome

The surgical procedure and the early postoperative period were uneventful. Follow‐up transthoracic echocardiography, performed 6 months later, revealed notable improvements in echocardiographic markers, including top‐normal right ventricular size with mild systolic dysfunction, no residual shunting, and a pulmonary‐to‐systemic ratio of 1.02. A routine follow‐up was, therefore, planned for the patient.

## 2 DISCUSSION

To the best of our knowledge, the present report is the first to introduce a rare association between UAPA (ALPA) and isolated PAPVC in an adult patient.

Unilateral absence of pulmonary artery, first described by Frentzel in 1868, is a rare disorder with a prevalence of 1 in 200 000 young adults; it can occur both in isolation and in association with other cardiovascular disorders.^4^, ^7^, ^8^, ^9^ The associated anomalies may include tetralogy of Fallot, atrial septal defect, coarctation of the aorta, right‐sided aortic arch, truncus arteriosus, and pulmonary atresia. Other disorders associated with UAPA are tracheal‐esophageal fistula; VACTERL (vertebral anomalies, anal atresia, cardiac malformations, Tracheo‐Esophageal fistula, renal anomalies, limb abnormalities) syndrome; gastrointestinal, musculoskeletal, and urogenital disorders; and chest and abdominal wall anomalies.^1^, ^10^, ^11^, ^12^

Embryologically, UAPA often occurs contralaterally to the aortic arch in consequence of the growth failure of the proximal sixth aortic arch segment in the presence of intact distal intrapulmonary branches, which are supplied through the bronchial, intercostal, internal mammary, subdiaphragmatic, subclavian, and even coronary arteries.^6^, ^13^, ^14^

In 2002, a study by Ten Harkel et al on 108 patients revealed that isolated UAPA was often diagnosed at an average age of 14 years. Thirty‐seven percent of the cases presented with symptoms such as chest pain, pleural effusion, and recurrent respiratory infections, and 40% of the cases showed symptoms of exercise intolerance. The authors reported that pulmonary hypertension complicated 44% of their patients.^2^

Two types of this disorder have been defined with regard to the time of symptom presentation: the infantile form, which presents with congestive heart failure and pulmonary hypertension, and the adult type, which is often asymptomatic.^15^, ^16^

In our patient, this disorder occurred in the uncommon form of adult‐type ALPA (type 3) with PAPVC (RUPV to SVC). To our knowledge, no association with PAPVC has been reported to date. The patient was almost asymptomatic despite ALPA with PAPVC until adulthood.

This anomaly is anatomically classified^17^ as type 1: agenesis, which is the complete absence of the bronchial tree, lung parenchyma, and ipsilateral pulmonary artery; type 2: aplasia, which is a complete lack of the lung parenchyma in the presence of a rudimentary bronchus; and type 3: hypoplasia, which is the presence of different amounts of the lung parenchyma, bronchial tree, and blood vessels.

The gold‐standard method for the diagnosis of this complication is angiography; nonetheless, advances in CT and CMR have rendered it unnecessary unless embolization is required due to massive hemoptysis.^5^

The most common symptoms of this disease are recurrent lung infections, decreased ability to work, and shortness of breath. Pulmonary infections are multifactorial and can be caused by decreased arterial blood flow, increased inflammatory cells, and ciliary dysfunction, leading to mucous trapping and bronchiectasis in some patients.^5^, ^6^ Hemoptysis is another manifestation of this disorder and is caused by increased collateral blood flow, which may progress to massive pulmonary hemorrhage and death.^18^, ^19^, ^20^ Hemoptysis and severe infections can be treated with embolization, lobectomy, or pneumonectomy.^21^, ^22^ However, any lung surgery in this disease may be complicated by the presence of collateral arteries.^6^

In infants with isolated absence of pulmonary artery, pulmonary hypertension is common because of the shear stress in the opposite‐side pulmonary artery, which leads to an increased release of vasoconstrictor factors such as endothelins. Chronic vasoconstriction results in remodeling, increased pulmonary vascular resistance, and pulmonary hypertension.^15^, ^16^

There is currently no specific treatment recommendation for UAPA. Some authors recommend serial echocardiography to monitor asymptomatic adult patients with a view to assessing pulmonary arterial pressure. Patients who develop pulmonary hypertension should be treated with vasodilators.^2^, ^23^ Alternatively, peripheral pulmonary branches can be focalized toward the pulmonary hilum. Successful revascularization procedures have been reported, especially in the pediatric population.^7^, ^9^

The prognosis of this disease is dependent on the associated cardiovascular anomalies and the severity of pulmonary hypertension. The overall reported mortality is 7%.^12^

In conclusion, UAPA is a rare congenital anomaly that involves the right‐sided lung in two‐thirds of this patient population. It consists of adult and infantile forms, with the former type remaining asymptomatic or minimally symptomatic. Advanced imaging modalities such as echocardiography, cardiac CT, and CMR have a pivotal role in the diagnosis of this anomaly. Rare associations are of interest and can be found in this disorder.

## CONFLICT OF INTEREST

None declared.

## AUTHOR CONTRIBUTIONS

Dr BJ and Dr LH: collected the data and prepared the primary draft, Dr NR and Dr SA: wrote the paper, and Dr SS: critically revised the article.

## ETHICAL APPROVAL

This study was approved by the Ethics Committee of Rajaie Cardiovascular Medical and Research Center, Iran University of Medical Sciences, Tehran, Iran.

## Data Availability

All data are available upon reasonable request.
